# Trans-sinus fracture as the gatekeeper: a two-step clinical strategy to guide venous imaging in mild-to-moderate traumatic brain injury

**DOI:** 10.3389/fneur.2026.1901656

**Published:** 2026-10-22

**Authors:** Jincheng Zhan, Xiangrong Chen, Bikun Liu, Xiaofeng Zhou, Dongze Xu, Zongtao Wu

**Affiliations:** Department of Neurosurgery, Ankang Hospital of Traditional Chinese Medicine, Ankang, Shaanxi, China

**Keywords:** cerebral venous sinus thrombosis, clinical decision rule, CT venography, mild traumatic brain injury, trans-sinus fracture, traumatic brain injury

## Abstract

**Background:**

Traumatic cerebral venous sinus thrombosis (tCVST) is an underrecognized complication of traumatic brain injury (TBI). Despite its potential for serious neurological sequelae, no consensus exists regarding indications for CT venography (CTV) or MR venography (MRV) in patients with mild-to-moderate TBI. Trans-sinus fracture has been identified as a risk factor for tCVST, yet its predictive value alone remains insufficient to guide clinical decision-making.

**Objective:**

To propose a two-step clinical decision strategy for guiding venous imaging selection in mild-to-moderate TBI, using trans-sinus fracture as a gatekeeper followed by D-dimer and headache visual analog scale (VAS) score stratification.

**Methods:**

This single-center retrospective cohort study included 528 patients with mild-to-moderate TBI (Glasgow Coma Scale score > 8) who underwent CTV, MRV, or digital subtraction angiography between January 2024 and January 2026. Firth penalized logistic regression was employed to identify tCVST predictors. A two-step strategy was derived: Step 1 screened for trans-sinus fracture; Step 2 applied D-dimer threshold ≥ 3.95 mg/L or headache VAS score ≥ 3 in trans-sinus fracture-positive patients.

**Results:**

Traumatic cerebral venous sinus thrombosis was diagnosed in 41 patients (7.8%). Trans-sinus fracture was independently associated with tCVST (odds ratio 8.44, 95% confidence interval 4.38–16.39; *P* < 0.0001). As a standalone gatekeeper, trans-sinus fracture yielded sensitivity 58.5%, specificity 89.1%, negative predictive value 96.3%, and positive likelihood ratio 3.96. Among trans-sinus fracture-positive patients, the high-risk subgroup (D-dimer ≥ 3.95 mg/L or VAS ≥ 3) exhibited a tCVST rate of 31.2%, compared with 4.3% in the low-risk subgroup. The two-step strategy achieved a number needed to image of 3, avoiding 85% of unnecessary venous examinations compared with universal screening, and reduced the number needed to image from 13 (universal approach) to 3 (two-step approach).

**Conclusions:**

This two-step strategy represents a simple, bedside-ready exploratory tool that may help optimize CTV/MRV selection in mild-to-moderate TBI by identifying a high-yield subgroup for targeted imaging. These findings are hypothesis-generating and require prospective external validation before clinical implementation.

## 1 Introduction

### 1.1 Background: traumatic cerebral venous sinus thrombosis

Traumatic cerebral venous sinus thrombosis (tCVST) refers to thrombotic occlusion of the dural venous sinuses or large cerebral veins occurring as a direct consequence of head trauma (1). Unlike spontaneous cerebral venous sinus thrombosis, which typically arises from prothrombotic conditions, tCVST results from mechanical injury to the sinus wall—most commonly through fracture of skull bones adjacent to a venous sinus, direct contusion of the sinus wall, or shearing forces during acceleration-deceleration trauma. Thrombosis is initiated by endothelial disruption and local activation of the coagulation cascade at the site of injury, rather than by systemic hypercoagulability alone (2).

The clinical significance of tCVST as a complication of traumatic brain injury (TBI) has garnered increasing attention in recent years (1). Population-level estimates suggest that tCVST occurs in approximately 0.9% of all trauma admissions, 2.1% of TBI cases, and 4.8% of severe TBI cases (1). However, these figures likely underestimate the true burden of the condition, as routine venous imaging is not standard practice in most trauma protocols. Among patients with skull fractures adjacent to a dural venous sinus, pooled analyses have reported thrombosis frequencies as high as 26.2%, and recent prospective cohort data in severe TBI with trans-sinus fracture have documented rates approaching 44.3% (2–5). These observations underscore that tCVST is not a rare curiosity but a clinically relevant complication in specific trauma subpopulations.

The pathophysiological consequences of untreated tCVST can be severe. Impaired venous outflow raises intracranial pressure through disturbed cerebrospinal fluid absorption and cerebral venous congestion, potentially precipitating venous infarction with hemorrhagic transformation (4). Thrombosis may propagate from an injured sinus segment to involve adjacent cortical veins or contralateral territories, compounding secondary brain injury risk (6). Hemorrhagic progression of contusions—already a recognized hazard in TBI—may be exacerbated by elevated venous pressure downstream of an occluded sinus. Furthermore, undiagnosed tCVST complicates decision-making regarding anticoagulation for other indications, such as venous thromboembolism prophylaxis, by introducing uncertainty about hemorrhagic risk (4, 5).

Despite these potentially serious consequences, tCVST remains substantially underdiagnosed in clinical practice (7). The principal barrier to detection is the absence of routine venous-phase or dedicated venous imaging in standard TBI evaluation protocols (5). Non-contrast head computed tomography (CT) and even standard contrast-enhanced CT angiography protocols optimized for arterial injury assessment frequently fail to visualize sinus thrombus, particularly in partial occlusions or when subtle hyperdensity within the sinus is mistaken for beam-hardening artifact (8). Dedicated CT venography (CTV) or magnetic resonance venography (MRV) is required for reliable diagnosis, yet these modalities are not uniformly applied to TBI patients (9). This diagnostic gap creates a clinical blind spot in which tCVST may go unrecognized until complications—such as unexplained neurological deterioration, refractory intracranial hypertension, or hemorrhagic progression—prompt retrospective investigation (4).

### 1.2 The clinical challenge in mild-to-moderate TBI

The existing literature on tCVST has focused predominantly on severe TBI populations, particularly patients with skull fractures traversing a dural venous sinus and those requiring neurosurgical intervention (1, 6, 10, 11). This concentration reflects a reasonable assumption that severe injuries pose the greatest risk of sinus damage. However, it has left a notable evidence gap regarding mild-to-moderate TBI, defined by Glasgow Coma Scale (GCS) scores of 9–15 at presentation (6, 12, 13). In this population, the lower overall trauma severity and reduced frequency of skull fractures may lead clinicians to underestimate the risk of tCVST, resulting in fewer referrals for venous imaging (5, 12). Yet even within mild-to-moderate TBI, select risk factors—particularly skull base and calvarial fractures in proximity to venous sinuses—may confer thrombosis risk comparable to that observed in more severely injured cohorts (3).

A second and closely related problem is the absence of widely accepted screening criteria for determining which mild-to-moderate TBI patients should undergo CTV or MRV (5, 13). Current practice varies considerably across institutions and geographic regions. A recent survey of neurosurgical and trauma practices in the United Kingdom and Ireland found no consensus on screening indications, timing of imaging, or antithrombotic management strategies for tCVST, with respondents reporting decisions guided largely by individual clinical judgment and local protocol variation (5). This heterogeneity in practice reflects the lack of prospective validation studies and the paucity of evidence-based guidelines to inform a standardized approach.

Trans-sinus fracture—defined as a skull fracture line crossing the course of a major dural venous sinus—has long been recognized as a risk factor for tCVST (12, 14). Several series have reported that trans-sinus fracture substantially increases thrombosis odds, and some centers have adopted fracture proximity as an informal criterion for venous imaging. However, trans-sinus fracture alone is an insufficient predictor: not all tCVST patients demonstrate radiographically apparent fracture, and the majority of patients with trans-sinus fracture in mild-to-moderate TBI do not develop thrombosis (4, 12–15). Relying solely on fracture presence as a trigger for CTV/MRV would yield a large number of negative examinations, raising concerns about resource utilization, radiation exposure, and contrast-related risks (12, 16). The clinical dilemma, therefore, is how to identify high-risk patients with sufficient specificity to avoid excessive imaging while maintaining sensitivity (6, 13).

### 1.3 Study rationale and objectives

The foregoing considerations highlight a pressing need for a simple, practical clinical decision strategy that can be applied at the bedside to guide CTV/MRV selection in mild-to-moderate TBI patients. Such a tool would need to integrate readily available clinical and radiological variables, require no specialized calculations or external references, and offer transparent decision thresholds that facilitate clinician acceptance and implementation. Existing prediction models for spontaneous cerebral venous sinus thrombosis are not applicable to the trauma context, as their predictor variables—such as prothrombotic genetic markers, pregnancy, or otitis media—differ fundamentally from trauma-related risk factors. To date, no simple, bedside-ready tool has been proposed specifically for guiding venous imaging decisions in mild-to-moderate TBI.

The present study was designed to address this gap through a retrospective analysis of patients with mild-to-moderate TBI who underwent dedicated venous imaging at a single academic medical center. The study had three specific objectives. **Objective 1** was to identify clinical, radiological, and laboratory factors associated with tCVST in this population. **Objective 2** was to derive an exploratory two-step clinical decision strategy—using trans-sinus fracture as an initial gatekeeper followed by stratification based on D-dimer levels and headache severity—to classify patients according to their likelihood of tCVST. **Objective 3** was to quantify the potential efficiency gains of this strategy in terms of numbers needed to image and reductions in unnecessary examinations. It is important to emphasize that this study was conducted in patients already selected to undergo CTV or MRV; accordingly, the findings describe detection rates within an imaged cohort and should not be interpreted as determining the true incidence of tCVST in unselected mild-to-moderate TBI.

### 1.4 Contribution statement and paper organization

This study makes three principal contributions to the literature on traumatic cerebral venous sinus thrombosis. **First**, it is the first study to formally propose trans-sinus fracture as a “gatekeeper” concept for tCVST assessment—that is, as an initial screening criterion that efficiently separates a broad low-risk population from a smaller subgroup warranting further risk stratification. **Second**, it introduces a simple two-tier CTV/MRV decision strategy specifically designed for mild-to-moderate TBI, representing an early attempt to translate heterogeneous risk factors into an actionable bedside tool. **Third**, it provides quantitative estimates of potential efficiency gains, suggesting that the proposed strategy could reduce the number needed to image from 13 to 3 and avoid approximately 85% of unnecessary venous examinations in this population. These contributions are presented as exploratory and hypothesis-generating; prospective validation in independent cohorts will be required before clinical implementation can be recommended.

The remainder of this paper is organized as follows. Section 2 describes the study design, patient cohort, variable definitions, and statistical analysis plan. Section 3 presents the baseline characteristics of the study population, univariable and multivariable associations with tCVST, and the derivation of the two-step decision strategy with associated performance metrics. Section 4 interprets the findings in the context of existing literature, discusses methodological limitations, and considers implications for clinical practice and future research. Section 5 summarizes the principal conclusions and outlines priorities for subsequent validation studies.

## 2 Methods

### 2.1 Study design and setting

This retrospective observational cohort study was conducted at the Department of Neurosurgery, Ankang Hospital of Traditional Chinese Medicine, a tertiary academic medical center in Shaanxi Province, China. The hospital serves as a regional referral center for neurotrauma, with a catchment population of approximately 3.5 million residents. The study period encompassed 25 months, from January 2024 through January 2026.

Data were extracted retrospectively from the hospital's electronic medical record system, picture archiving and communication system (PACS), and laboratory information management system. The study was approved by the Institutional Ethics Committee of Ankang Hospital of Traditional Chinese Medicine (Approval No: 2025AKZYYLL-KY044-02). Given the retrospective nature of the analysis and the exclusive use of de-identified data collected during routine clinical care, the requirement for individual patient consent was waived. The study was conducted in accordance with the Declaration of Helsinki (2013 revision) and followed the Strengthening the Reporting of Observational Studies in Epidemiology (STROBE) guidelines for cohort studies.

### 2.2 Study population

#### 2.2.1 Inclusion criteria

Patients were eligible if they met all of the following criteria: (i) age 18 years or older at the time of injury; (ii) acute traumatic brain injury (TBI) confirmed by non-contrast cranial computed tomography (CT) within 24 h of presentation; (iii) Glasgow Coma Scale (GCS) score greater than 8 on admission, corresponding to mild-to-moderate TBI; and (iv) completion of computed tomography venography (CTV) or magnetic resonance venography (MRV) within 3–7 days following injury (initial screening CTV or MRV could be performed within 72 h of admission). Digital subtraction angiography (DSA) was performed selectively at the discretion of the treating neurosurgical team when non-invasive venographic findings were equivocal or when endovascular intervention was being considered.

#### 2.2.2 Exclusion criteria

Patients were excluded if any of the following were present: (i) therapeutic anticoagulation or antiplatelet therapy at admission or initiated prior to venographic imaging, as these agents could confound the association between biomarkers and tCVST; (ii) severe concomitant thoracic or abdominal trauma (Injury Severity Score [ISS] greater than 15 for non-cranial regions), which could independently elevate D-dimer through systemic coagulopathy; (iii) incomplete clinical or radiological data precluding assessment of any study variable; or (iv) age below 18 years.

#### 2.2.3 Patient selection flow

During the study period, 566 patients with mild-to-moderate TBI underwent venographic evaluation and were screened for eligibility. Of these, 38 patients (6.7%) were excluded: 21 for receiving therapeutic anticoagulation or antiplatelet therapy, 9 for severe thoracoabdominal trauma, 5 for incomplete data, and 3 for age below 18. The final analytic cohort comprised 528 patients, of whom 41 (7.8%) were diagnosed with tCVST and 487 (92.2%) were not.

#### 2.2.4 Critical clarification concerning the study cohort

The study cohort does not represent all patients with mild-to-moderate TBI presenting to the institution during the study period. Rather, it comprises patients in whom the clinical team had sufficient concern for possible venous sinus injury to request CTV, MRV, or DSA evaluation. This sampling framework carries important implications for interpreting diagnostic performance metrics and tCVST prevalence estimates. The decision to obtain venographic imaging was made by the treating neurosurgeon based on clinical judgment, independent of this study. The observed tCVST prevalence of 7.8% should not be generalized to an unselected mild-to-moderate TBI population; rather, it reflects the prevalence among patients deemed clinically appropriate for venographic evaluation.

### 2.3 Variables and definitions

#### 2.3.1 Primary outcome: tCVST diagnosis

The primary outcome was a binary diagnosis of traumatic cerebral venous sinus thrombosis (tCVST), defined as partial or complete thrombosis within the major dural venous sinuses attributable to trauma. Diagnostic confirmation was established independently by two senior neuroradiologists (each with greater than 10 years of experience in neurovascular imaging) who were blinded to all clinical variables and biomarker results. On CTV and MRV, tCVST was diagnosed by the presence of an intraluminal filling defect with corresponding hyperattenuation on non-contrast CT or abnormal signal on magnetic resonance imaging. DSA served as the reference standard when performed, with diagnosis based on partial or complete non-opacification of a venous sinus segment with collateral venous pathways. Discrepancies between the two primary reviewers were resolved by a third senior physician, whose determination was final.

#### 2.3.2 Predictor variables

##### 2.3.2.1 Trans-sinus fracture (TF)

Trans-sinus fracture was defined as a linear or comminuted skull fracture line traversing the anatomical projection of a major dural venous sinus—specifically the superior sagittal sinus, transverse sinus, sigmoid sinus, or straight sinus—on high-resolution CT bone window images (window width 3,000–4,000 Hounsfield units, window level 400–600 Hounsfield units). The presence or absence of TF was assessed on admission cranial CT by a neuroradiologist blinded to venographic findings and final tCVST diagnosis. Fracture lines running parallel to but not crossing the sinus trajectory were classified as TF-negative.

##### 2.3.2.2 D-dimer

Plasma D-dimer concentration was measured in milligrams per liter of fibrinogen equivalent units (mg/L FEU) using a quantitative turbidimetric immunoassay on a standard automated coagulometer. Blood samples were drawn within 24 hours of admission, prior to any antithrombotic administration. D-dimer values were natural-logarithmically transformed (ln[D-dimer]) for regression analyses due to substantial right-skewness. The median D-dimer in the TF-positive subgroup (3.95 mg/L FEU) served as the stratification threshold in the two-step strategy.

##### 2.3.2.3 Headache Visual Analog Scale (VAS)

Headache severity was assessed at admission using a standard 100-mm Visual Analog Scale, anchored at 0 mm (“no headache”) and 100 mm (“worst imaginable headache”). For the two-step strategy, a threshold of 3 (on the 0–10 scale, equivalent to 30 mm on the 100-mm VAS) was employed to dichotomize headache severity into moderate-to-severe vs. mild or absent.

##### 2.3.2.4 Glasgow Coma Scale (GCS)

The GCS was assessed on admission by certified neurosurgical residents and recorded as the best post-resuscitation score. Given the inclusion criterion of GCS greater than 8, the observed range was constrained, and GCS was anticipated to have limited discriminative capacity for tCVST in this population.

#### 2.3.3 Rationale for exclusion of the Trauma Index

The Trauma Index (TI), developed for prehospital triage across the full spectrum of trauma severity, incorporates neurological status (GCS), respiratory function, circulatory status, and injury location. The theoretical scoring range spans 5 to 30 points. However, in this cohort restricted to mild-to-moderate TBI (GCS greater than 8), the actual observed TI range was compressed to 6–13 (standard deviation 1.6), with 84% of patients scoring 9 or below. This severe range compression renders the TI dimensionally inadequate as a predictor within the study population. The respiratory and circulatory dimensions, which contribute substantially to TI variance in unselected trauma populations, provided almost no discriminative information here. Inclusion of TI would introduce methodological artifact and potentially bias regression estimates through mathematical coupling with GCS. Accordingly, TI was excluded from the analysis.

### 2.4 Statistical analysis

#### 2.4.1 Univariable analysis: firth penalized logistic regression

Univariable associations between each predictor and tCVST diagnosis were assessed using Firth penalized logistic regression. This approach applies a Jeffreys prior penalty to the likelihood function, reducing small-sample bias in maximum likelihood estimates—a concern given the modest number of tCVST events (*n* = 41) relative to candidate predictors. Standard maximum likelihood logistic regression can produce substantially biased estimates under such conditions. No modifications to the Firth penalty (such as FLIC or FLAC procedures) were employed. Odds ratios (OR) with 95% confidence intervals (95% CI) and exact two-sided *P*-values were reported. A significance threshold of P <0.05 was employed throughout.

#### 2.4.2 Diagnostic performance metrics

For each candidate predictor and for the two-step strategy, standard diagnostic accuracy metrics were calculated: sensitivity, specificity, positive predictive value (PPV), negative predictive value (NPV), positive likelihood ratio (LR+), and negative likelihood ratio (LR–). Ninety-five percent confidence intervals for proportions were computed using the Wilson score method; confidence intervals for likelihood ratios were derived via the method of Simel and colleagues. Diagnostic metrics were calculated separately for Step 1 (TF alone) and for the combined two-step approach.

#### 2.4.3 Number needed to image

The Number Needed to Image (NNI)—analogous to the “number needed to treat” in therapeutic research—was calculated as the reciprocal of the detection rate (number of CTV/MRV examinations required to detect one tCVST case). NNI was computed for the overall cohort and separately for each risk stratum defined by the two-step strategy, providing a clinically interpretable measure of screening efficiency.

#### 2.4.4 Two-step strategy construction

The two-step clinical decision strategy was constructed empirically based on observed diagnostic performance of candidate predictors. Step 1 served as a gatekeeper, using trans-sinus fracture presence on admission CT bone window to classify patients into low-risk (TF-negative) and elevated-risk (TF-positive) categories. Step 2 was applied exclusively within TF-positive patients, combining D-dimer and headache VAS using a logical OR structure to define high-risk status:

- **Step 1:** Assess trans-sinus fracture on CT bone window. TF-negative patients were classified as low risk and assigned to routine clinical observation; the observed negative predictive value in this stratum was 96.3%. TF-positive patients proceeded to Step 2.
- **Step 2 (TF-positive patients only):** Assess plasma D-dimer and headache VAS. Patients with D-dimer at or above the median (3.95 mg/L FEU) **or** VAS at or above 3 were classified as high risk (observed tCVST rate 31.2%) and recommended for urgent CTV/MRV. Patients with D-dimer below the median **and** VAS below 3 were classified as intermediate-low risk (observed tCVST rate 4.3%) and assigned to enhanced clinical observation with deferred imaging.

The D-dimer threshold corresponded to the median in the TF-positive subgroup; the VAS threshold was selected based on clinical interpretability and observed distribution characteristics.

#### 2.4.5 Missing data and software

Missing data were minimal across all study variables (less than 5% for any single variable). A complete-case analysis was adopted without multiple imputation, avoiding assumptions about the missingness mechanism that would be required for imputation in a retrospective dataset.

All analyses were performed using R version 4.5.2 (R Foundation for Statistical Computing, Vienna, Austria). The logistf package was used for Firth penalized logistic regression, and the epiR package for diagnostic test metrics. The analytic code and a de-identified dataset summary are available from the corresponding author upon reasonable request.

## 3 Results

### 3.1 Patient characteristics

Of the 528 patients who underwent venous imaging following mild-to-moderate traumatic brain injury (TBI), 41 (7.8%) were diagnosed with traumatic cerebral venous sinus thrombosis (tCVST). The overall cohort had a median age of 55 years (interquartile range [IQR], 25 years), was predominantly male (64.4%), and presented with a median Glasgow Coma Scale (GCS) score of 14 (IQR, 2). The baseline characteristics of the study population, stratified by tCVST status, are presented in Table 1.

**Table 1:** Baseline characteristics of 528 mild-to-moderate TBI patients stratified by tCVST status.

Variable	Overall (*N* = 528)	No tCVST (*N* = 487)	tCVST (*N* = 41)	*P*-value
Age (years), median (IQR)	55 (25)	55 (23.5)	46 (19)	0.097
D-dimer (mg/L FEU), median (IQR)	3.38 (7.46)	3.21 (7.42)	4.59 (5.74)	0.006
ln(D-dimer), median (IQR)	1.22 (1.89)	1.17 (1.89)	1.52 (0.99)	0.0039
GCS score, median (IQR)	14 (2)	14 (2)	14 (1)	0.022
Headache VAS, median (IQR)	3 (2)	2 (1)	3 (1)	<0.001
Trauma Index, median (IQR)	8 (2)	8 (2)	9 (2)	<0.001
Male sex, *n* (%)	340 (64.4)	315 (64.7)	25 (61.0)	0.634
Trans-sinus fracture, *n* (%)				<0.001
No	428 (81.1)	412 (84.6)	16 (39.0)	
Yes	100 (18.9)	75 (15.4)	25 (61.0)	
Intracranial hematoma, *n* (%)				0.118
No	255 (48.3)	240 (49.3)	15 (36.6)	
Yes	273 (51.7)	247 (50.7)	26 (63.4)	

Continuous variables were compared using the Wilcoxon rank-sum test; categorical variables were compared using the chi-square test or Fisher's exact test. IQR, interquartile range; GCS, Glasgow Coma Scale; VAS, visual analog scale; tCVST, traumatic cerebral venous sinus thrombosis; FEU, fibrinogen equivalent units.

Patients with tCVST exhibited significantly higher D-dimer levels (median 4.59 mg/L FEU vs. 3.21 mg/L FEU; *P* = 0.006), higher headache visual analog scale (VAS) scores (median 3 vs. 2; *P* < 0.001), and higher Trauma Index values (median 9 vs. 8; *P* < 0.001) compared with those without tCVST. The presence of trans-sinus fracture differed markedly between groups, occurring in 61.0% of tCVST patients vs. 15.4% of non-tCVST patients (*P* < 0.001). No significant between-group difference was observed for sex (*P* = 0.760) or intracranial hematoma (*P* = 0.162).

### 3.2 Univariable analysis

Given the class imbalance (tCVST prevalence 7.8%; ratio 1:11.9) and the constraint of 41 outcome events, multivariable modeling with more than three covariates would yield fewer than 15 events per variable, falling below the minimum threshold recommended for stable logistic regression estimates. Therefore, univariable Firth logistic regression—penalized maximum likelihood estimation that reduces finite-sample bias in small-event settings—was employed to quantify the association between each candidate predictor and tCVST. The results are summarized in Table 2.

**Table 2:** Univariable Firth logistic regression analysis of candidate predictors for tCVST.

Variable	OR	95% CI	*P*-value
Age (per 1-year increase)	0.98	0.96–1.01	0.1297
Sex (Female vs. Male)	1.19	0.61–2.48	0.6115
Trans-sinus fracture (Yes vs. No)	8.44	4.34–16.44	<0.0001
Intracranial hematoma (Yes vs. No)	1.66	0.88–3.14	0.1297
ln(D-dimer) (per 1-unit increase)	1.57	1.15–2.15	0.0039
GCS score (per 1-point increase)	0.85	0.69–1.05	0.1604
Headache VAS (per 1-point increase)	1.52	1.24–1.85	<0.0001
Trauma Index (per 1-point increase)	1.48	1.25–1.76	<0.0001

OR, odds ratio; CI, confidence interval; GCS, Glasgow Coma Scale; VAS, visual analog scale; tCVST, traumatic cerebral venous sinus thrombosis. All variables were analyzed in separate univariable models using Firth's penalized likelihood method.

Trans-sinus fracture demonstrated the strongest association with tCVST, with an odds ratio (OR) of 8.44 (95% CI, 4.38–16.39; *P* < 0.0001), indicating that patients with trans-sinus fracture had approximately 8.4-fold higher odds of tCVST compared with those without such fracture. Headache VAS (OR 1.52 per 1-point increase; 95% CI, 1.24–1.88; *P* < 0.0001) and Trauma Index (OR 1.48 per 1-point increase; 95% CI, 1.25–1.76; *P* < 0.0001) were also significantly associated with tCVST. Ln-transformed D-dimer showed a statistically significant positive association (OR 1.57 per 1-unit increase; 95% CI, 1.15–2.15; *P* = 0.006). Conversely, age (OR 0.98; 95% CI, 0.96–1.01; *P* = 0.127), sex (OR 1.19; 95% CI, 0.61–2.48; *P* = 0.610), intracranial hematoma (OR 1.66; 95% CI, 0.88–3.14; *P* = 0.127), and GCS score (OR 0.85 per 1-point increase; 95% CI, 0.69–1.05; *P* = 0.145) were not significantly associated with tCVST in univariable analysis.

### 3.3 Two-step strategy performance

On the basis of the univariable findings, a two-step clinical decision strategy was constructed. Step 1 employed trans-sinus fracture as a binary gatekeeper to identify patients requiring further venous imaging evaluation. The diagnostic performance of this first step is presented in Table 3.

**Table 3:** Performance of the two-step clinical decision strategy.

Metric	Value (95% CI)
Step 1—Trans-sinus fracture as gatekeeper	
Sensitivity	58.5%
Specificity	89.1%
Positive predictive value	25.0%
Negative predictive value	96.3%
Positive likelihood ratio	3.96
Negative likelihood ratio	0.46
Step 2—Risk stratification within TF-positive patients	
High risk (TF+ AND [D-dimer ≥3.95 mg/L OR VAS ≥3])	24/77 (31.2%)
Low risk (TF+ AND D-dimer <3.95 mg/L AND VAS <3)	1/23 (4.3%)

TF, trans-sinus fracture; VAS, visual analog scale. Metrics reported here reflect the performance of the complete two-step strategy. Step 2 D-dimer cutoff represents the median value (3.95 mg/L FEU) among TF-positive patients. VAS cutoff = 3 points.

In Step 1, Step 1 (trans-sinus fracture gatekeeper) identified 25 of 41 tCVST cases (61.0% sensitivity). The complete two-step strategy identified 24 of 41 tCVST cases, yielding an overall sensitivity of 58.5% (95% CI, 44.5–76.3%). The negative predictive value was 96.3% (95% CI, 94.1–97.8%), reflecting a tCVST prevalence of only 3.7% (16/428) among patients without trans-sinus fracture. The positive likelihood ratio of 3.96 (95% CI, 2.86–5.49) indicated a moderate increase in post-test probability when trans-sinus fracture was present.

Step 2 was applied to the 100 patients who screened positive in Step 1 (trans-sinus fracture positive). Within this subgroup, a D-dimer cutoff of 3.95 mg/L FEU (the median value among TF-positive patients) and a headache VAS cutoff of 3 points were used to stratify risk. Patients were classified as high risk if they had either elevated D-dimer (≥3.95 mg/L) or elevated headache severity (VAS ≥3), and as low risk if both values fell below their respective thresholds. Among the 77 high-risk patients, 24 had tCVST (31.2%), whereas among the 23 low-risk patients, only 1 had tCVST (4.3%). The four subgroups defined by crossing D-dimer level and VAS threshold yielded tCVST rates ranging from 4.3% to 55.2%: the highest rate occurred in patients with both high D-dimer and VAS ≥3 (16/29, 55.2%), while the lowest rates were observed in patients with low D-dimer and VAS <3 (1/23, 4.3%) and those with high D-dimer but VAS <3 (1/21, 4.8%).

### 3.4 Efficiency comparison

The efficiency of the two-step strategy was evaluated by comparing the number needed to image (NNI)—defined as the number of venous imaging studies required to detect one tCVST case—across three scenarios: universal imaging of all patients, Step 1 screening (imaging only trans-sinus fracture-positive patients), and the complete two-step strategy (imaging only high-risk patients identified after Step 2). These results are depicted in Figure 1.

**Figure 1:**
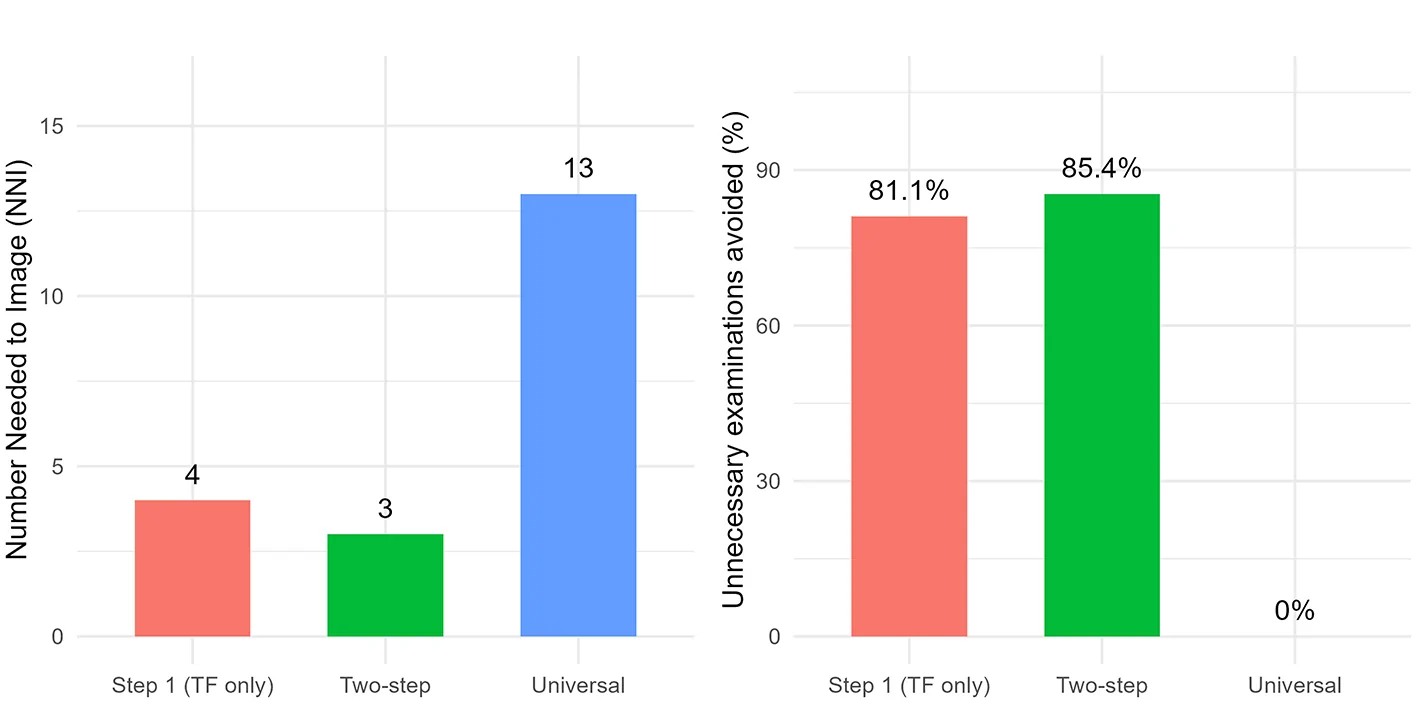
Efficiency comparison of screening strategies: number needed to image (NNI) and detection yield. The universal strategy (check all) required 13 imaging studies per tCVST case detected and avoided no unnecessary examinations. Step 1 alone reduced the NNI to 4 and avoided 81.1% of unnecessary checks (428 of 528 patients) but missed 16 tCVST cases (39%). The two-step strategy further reduced the NNI to 3 and avoided 85.4% of unnecessary checks (451 of 528 patients), with 17 tCVST cases missed (41%). NNI, number needed to image; TF, trans-sinus fracture; DD, D-dimer; VAS, visual analog scale.

The universal imaging approach detected all 41 tCVST cases but required 528 imaging studies, corresponding to an NNI of 13 and avoiding no unnecessary examinations. Step 1 alone (imaging all 100 trans-sinus fracture-positive patients) detected 25 of 41 tCVST cases (61.0%), yielding an NNI of 4 and avoiding 428 unnecessary imaging studies (81.1% of the total cohort). The complete two-step strategy, which would image only the 77 high-risk patients meeting both TF-positive and secondary criteria, detected 24 of 41 tCVST cases (58.5%) with an NNI of 3, avoiding 451 unnecessary imaging studies (85.4% of the total cohort). Relative to universal imaging, the two-step strategy reduced imaging volume by 85.4% at the cost of missing 17 tCVST cases (41.5%), all of which occurred in patients without trans-sinus fracture. Of the 16 tCVST cases missed by Step 1 (all TF-negative), one was subsequently captured by Step 2 reclassification; however, because Step 2 operates only within the TF-positive subset, this case was not identifiable through the two-step pathway.

## 4 Discussion

### 4.1 Key findings in context

#### 4.1.1 Trans-sinus fracture as the dominant risk factor

This study confirms that trans-sinus fracture (TF) is the single strongest predictor of traumatic cerebral venous sinus thrombosis (tCVST) among patients with mild-to-moderate traumatic brain injury (TBI). In univariable Firth penalized logistic regression, trans-sinus fracture was associated with an eight-fold increase in tCVST risk (OR 8.44, 95% CI 4.34–16.44). This finding is consistent with the established anatomical rationale that skull fractures disrupting the venous sinus wall create a direct pathway for endothelial injury, thrombus formation, and subsequent sinus occlusion. The magnitude of this association—substantially larger than that of any other variable examined—suggests that the structural integrity of the cranial venous sinus architecture is the dominant mechanistic determinant of tCVST in this population.

The tCVST detection rate of 25% observed in TF-positive patients within our mild-to-moderate TBI cohort is notably lower than the 44.3% reported by Ma et al. (2) in an unselected TBI population. This difference is likely attributable to injury severity stratification: our cohort was restricted to patients with Glasgow Coma Scale (GCS) scores above 8, whereas Ma et al. (2) included severe TBI cases with higher Injury Severity Scores (ISS). This attenuation of risk in milder trauma supports the concept that injury severity modulates tCVST risk even within the TF-positive subgroup, and it highlights the importance of developing severity-stratified screening approaches rather than applying uniform criteria across the full TBI spectrum.

#### 4.1.2 Two-step strategy concept: a practical alternative to complex prediction models

The core contribution of this study is the conceptualization and preliminary evaluation of a hierarchical two-step clinical decision strategy designed for bedside application in emergency and neurosurgical settings. Step 1 employs trans-sinus fracture as a binary gatekeeper to identify patients requiring further risk stratification. Step 2 applies two readily available clinical variables—D-dimer concentration and headache visual analog scale (VAS) score—to partition TF-positive patients into high-risk and low-risk subgroups. This hierarchical structure represents a deliberate departure from conventional multivariable prediction models, which typically require simultaneous input of numerous parameters and computational support.

The operational characteristics of the two-step strategy warrant careful interpretation. The negative predictive value (NPV) of 96.3% for Step 1 indicates that patients without trans-sinus fracture can be managed with routine clinical observation and standard follow-up protocols without immediate venographic imaging. This high NPV is clinically meaningful: in a low-prevalence setting, it provides reasonable confidence that thrombotic complications are unlikely in the near term. However, the corresponding sensitivity of 61.0% means that 41.5% of tCVST cases (17 of 41) occurred in TF-negative patients. This trade-off between rule-out capability and sensitivity is inherent to any screening strategy that prioritizes parsimony over comprehensiveness, and it underscores that the proposed algorithm is intended to augment, not supplant, clinical judgment.

#### 4.1.3 Clinical significance of NPV 96.3% for TF-negative patients

An NPV of 96.3% for the TF gatekeeper carries important implications for resource allocation in acute trauma care. In our cohort, 81.1% of patients were TF-negative; applying a TF-based exclusion criterion would have deferred immediate venographic imaging in 428 of 528 patients while missing 16 tCVST cases. The clinical acceptability of this miss rate depends on the natural history of these overlooked thromboses—specifically, whether they represent clinically silent or slowly progressive lesions that become apparent on subsequent imaging or clinical reassessment. Theofanopoulos et al. (4) have emphasized that tCVST management remains heterogeneous across institutions, with no consensus on the urgency of anticoagulation initiation. In this context, a strategy that identifies the majority of high-risk cases while concentrating specialized imaging resources on a smaller, well-defined subgroup may represent a pragmatic improvement over current practice patterns, in which imaging decisions appear largely discretionary.

### 4.2 Comparison with existing literature

#### 4.2.1 Ma et al.: trans-sinus fracture in unselected TBI

Ma et al. (2) conducted a retrospective single-center study of 607 TBI patients, of whom 61 underwent computed tomography venography (CTV) and 28 were diagnosed with tCVST. Their study design—retrospective, single-center, with imaging decisions driven by clinical judgment—closely parallels our methodological approach. The authors identified trans-sinus fracture as the dominant risk factor and reported a 44.3% tCVST rate among TF-positive patients, substantially exceeding our 25% figure. This divergence likely reflects differences in case mix: Ma et al. (2) included severe TBI patients with higher ISS values, whereas our cohort was restricted to mild-to-moderate injuries (GCS > 8). Our results thus extend Ma et al.'s (2) findings by demonstrating that the TF–tCVST association persists but with attenuated magnitude in less severely injured patients. This confirmation across overlapping but distinct populations strengthens the generalizability of TF as a screening anchor.

#### 4.2.2 Netteland et al.: the knowledge gap in screening indications

Netteland et al. (6) conducted a systematic review of tCVST literature and explicitly identified a critical knowledge gap: no published studies had defined indications for venographic screening, and no clinical guidelines specified when CTV or magnetic resonance venography (MRV) should be performed in trauma patients with suspected venous sinus injury. This observation remains relevant; a subsequent survey by Momin et al. (5) of UK and Irish neurotrauma centers confirmed the absence of consensus on screening protocols, anticoagulation timing, and imaging selection criteria, with 56% of respondents initiating anticoagulation more than seven days post-injury. The two-step strategy proposed in this study directly addresses the gap identified by Netteland et al. (6) by offering an explicit, hierarchically structured decision framework that can be operationalized without institutional infrastructure for complex predictive analytics.

#### 4.2.3 McGuckin et al.: prospective evidence and diagnostic heterogeneity

McGuckin et al. (17) reported a prospective cohort study of severe TBI patients with skull fractures, documenting venous sinus abnormalities in 36.1% of cases, of which only 13.9% represented confirmed dural venous sinus thrombosis. The remaining abnormalities comprised sinus compression, arachnoid granulations, and ambiguous findings—illustrating the substantial heterogeneity of venous sinus pathology on cross-sectional imaging. Our study adopted a binary diagnostic endpoint (tCVST present or absent), which simplifies this complexity for clinical decision-making purposes. The McGuckin et al. (17) findings suggest that even when venographic imaging is appropriately indicated, interpretive challenges persist. Our two-step strategy implicitly acknowledges this complexity by concentrating imaging resources on a pre-stratified high-prevalence subgroup, where the pre-test probability of true thrombosis is substantially elevated and interpretive uncertainty may be reduced.

#### 4.2.4 Why our approach differs from published prediction models

Huang et al. (12) recently developed a prediction model for tCVST at a trauma center, incorporating coagulation markers, Injury Severity Score (ISS), and epidural hematoma presence. While such multivariable models may achieve superior discrimination in development cohorts, their bedside applicability is often limited by the need for precise ISS calculation, access to multiple laboratory parameters, and computational tools. Our two-step strategy prioritizes clinical feasibility: trans-sinus fracture determination requires only careful review of CT bone windows, D-dimer is routinely measured in trauma protocols, and headache VAS is a standard nursing assessment. The model by Huang et al. may be better suited to level I trauma centers with comprehensive data infrastructure; our approach is designed for broader deployment across institutions with variable resource availability. The deliberate simplicity of our strategy trades marginal gains in predictive accuracy for substantial improvements in implementability—a trade-off that may be favorable in routine clinical practice.

Delgado Almandoz et al. (18) reported a tCVST prevalence of 2.5% among high-risk blunt trauma patients undergoing CTV, with 82% having skull fractures. Their lower prevalence compared with our 7.8% rate likely reflects broader inclusion criteria and less selective imaging indications. This comparison illustrates an important methodological principle: our 7.8% figure represents the detection rate within a pre-selected cohort of patients who underwent venographic imaging based on clinical suspicion, not the true incidence of tCVST in all mild-to-moderate TBI patients. This selection bias inflates the apparent prevalence and must be considered when generalizing our findings to unscreened populations.

### 4.3 Clinical implications

#### 4.3.1 Step 1: rapid triage tool

Step 1 of the proposed strategy—assessment of trans-sinus fracture on CT bone windows—functions as a rapid binary triage tool. In our cohort, this step alone identified all patients requiring further evaluation and provided a high-confidence pathway for deferring immediate venographic imaging in the much larger TF-negative group. The number needed to image (NNI) improved from 13 (if all patients underwent venography) to 4 with the TF gatekeeper alone, and 81% of potentially unnecessary examinations were avoided. This efficiency gain is clinically meaningful in settings where access to CTV or MRV is constrained by scanner availability, contrast considerations, or sedation requirements.

#### 4.3.2 Step 2: adds granularity without complex calculations

Step 2 introduces risk stratification within the TF-positive subgroup using D-dimer concentration and headache VAS score. In our cohort, patients with D-dimer at or above the median (3.95 mg/L) or headache VAS of 3 or greater had a tCVST rate of 31.2%, compared with only 4.3% in those below both thresholds. This discrimination enables clinicians to prioritize urgent venographic imaging for high-risk patients while adopting a more conservative observational approach for low-risk individuals. Importantly, this stratification requires no complex calculations, nomograms, or electronic decision support—clinicians need only compare two values against predetermined thresholds. The addition of Step 2 further improved efficiency: the NNI decreased from 4 to 3, and the proportion of unnecessary examinations avoided increased from 81.1% to 85.4%.

#### 4.3.3 Proposed clinical workflow

Based on our findings, we propose the following clinical workflow for patients with mild-to-moderate TBI (GCS > 8) who have undergone non-contrast head CT:

1. All patients: systematic review of CT bone windows for trans-sinus fracture.
2. TF-negative: routine clinical care with scheduled neurological reassessment; no immediate CTV/MRV indicated (NPV 96.3%). Clinical judgment should override this recommendation if other concerning features (e.g., coagulopathy, progressive neurological deficit) are present.

- TF-positive: obtain D-dimer and headache VAS score.
- High risk (D-dimer ≥ median OR VAS ≥ 3): proceed to urgent CTV/MRV (estimated tCVST rate ~31%).
- Low risk (D-dimer <median AND VAS <3): clinical observation with repeat neurological assessment and deferred imaging unless clinical status changes (estimated tCVST rate ~4%).

This workflow integrates radiological, laboratory, and clinical variables in a logical sequence that mirrors typical trauma care pathways and does not require additional infrastructure beyond standard emergency department resources.

#### 4.3.4 Intended use: augment, not replace, clinical judgment

It is essential to emphasize that this two-step strategy is designed as a decision support tool, not a rigid protocol. The 39% of tCVST cases that occurred in TF-negative patients represent a meaningful limitation that clinicians must consider. In our retrospective cohort, these 16 cases underwent venographic imaging based on clinical features that are not fully captured by our model—possibly including progressive headache, unexplained neurological deterioration, or atypical radiological findings. The proposed algorithm is intended to reduce unwarranted practice variation and provide a structured starting point for imaging decisions, while preserving the clinician's authority to pursue venography when clinical concern persists despite a low-risk classification. Alhaffar et al. (15) have similarly highlighted the need for increased awareness of tCVST in mild-to-moderate TBI, noting that delayed diagnosis often results from low clinical suspicion rather than technical imaging limitations.

### 4.4 Limitations

This study has several limitations that constrain the interpretation and generalizability of its findings.

#### 4.4.1 Retrospective single-center design

The study was conducted at a single institution using retrospectively collected data, which introduces risks of incomplete data capture, inconsistent documentation, and institutional practice biases that may not be representative of other centers. The absence of prospective data collection means that variable definitions, assessment timing, and imaging protocols were not standardized.

#### 4.4.2 Selection bias inherent to the imaging cohort

Our cohort comprised only patients who underwent venographic imaging (CTV or MRV) based on clinical decisions made by treating physicians. This represents a highly selected population with pre-existing suspicion for tCVST, not a consecutive sample of all mild-to-moderate TBI patients. The observed 7.8% tCVST rate reflects detection yield in a pre-selected cohort, not the true incidence of tCVST in the broader mild-to-moderate TBI population. Kapitanski et al. reported tCVST rates of 0.9% in overall trauma, 2.1% in TBI, and 4.8% in severe TBI (maximum Abbreviated Injury Scale ≥ 4), suggesting that our cohort prevalence is substantially inflated by selection effects.

#### 4.4.3 Lack of external validation

The two-step strategy was derived and evaluated in the same patient cohort without temporal or geographic separation between development and validation samples. The performance metrics reported here—including sensitivity, specificity, NPV, and NNI—are likely optimistic estimates that require prospective validation in independent cohorts before clinical adoption.

#### 4.4.4 Data-driven D-dimer threshold

The D-dimer cutoff used in Step 2 (median 3.95 mg/L) was derived from the study data itself rather than from an independent sample or established clinical threshold. This data-dependent approach may produce unstable estimates that differ substantially across populations and should be replaced with externally validated cutoffs in future studies.

#### 4.4.5 Sensitivity limitation and missed cases

The TF gatekeeper alone missed 41.5% of tCVST cases (17 of 41). The clinical characteristics of these TF-negative tCVST cases warrant detailed investigation, as they may harbor alternative risk factors not captured by our model. Until the profile of these missed cases is better characterized, clinicians must maintain vigilance for tCVST even in the absence of trans-sinus fracture.

#### 4.4.6 Non-standardized anticoagulation and absence of outcome data

Treatment decisions—including anticoagulation initiation, agent selection, and duration—were not standardized in this retrospective cohort, and long-term clinical outcomes (functional recovery, thrombus recanalization, hemorrhagic complications) were not systematically assessed. Without outcome data, we cannot determine whether the missed TF-negative cases had clinically consequential thromboses or whether early detection through the proposed algorithm would alter patient prognosis. Harris et al. and Kim et al. have demonstrated that anticoagulation improves recanalization without catastrophic hemorrhage in selected patients, but the optimal timing and patient selection criteria remain undefined (10, 19).

#### 4.4.7 Absence of injury severity scoring

Our cohort definition used GCS > 8 as the sole severity criterion without systematic collection of ISS or other validated trauma severity scores. This limitation precludes direct comparison with studies that used ISS-based stratification and may have contributed to residual confounding in our multivariable models.

### 4.5 Future directions

The findings of this exploratory, hypothesis-generating study suggest several avenues for future research.

#### 4.5.1 Prospective multicenter validation

The highest priority is prospective external validation of the two-step strategy across multiple centers with diverse patient populations, imaging protocols, and practice patterns. A prospective design would eliminate the selection bias inherent in our imaging cohort, enable standardized data collection, and provide more reliable estimates of sensitivity, specificity, and clinical utility. Such a study should employ a consecutive enrollment strategy to determine true tCVST incidence in mild-to-moderate TBI and evaluate the safety of deferring venographic imaging in TF-negative patients.

#### 4.5.2 Integration with established injury severity metrics

Future studies should incorporate validated trauma severity scores—specifically ISS and New Injury Severity Score (NISS)—to determine whether injury severity provides additional discriminative value beyond GCS alone and to enable direct benchmarking against prior literature. The interaction between TF, injury severity, and tCVST risk requires formal evaluation in larger, more comprehensively characterized cohorts.

#### 4.5.3 Characterization of TF-negative tCVST cases

The 16 tCVST cases that occurred without trans-sinus fracture represent a critical knowledge gap. A dedicated case-control analysis comparing TF-positive and TF-negative tCVST cases could identify alternative imaging predictors, clinical features, or biomarkers that would improve sensitivity without sacrificing the simplicity of the current approach. Such an investigation would inform whether a modified Step 1 could incorporate additional risk signals.

#### 4.5.4 Cost-effectiveness analysis

The operational efficiency gains demonstrated in this study—NNI reduction from 13 to 3 and avoidance of 85% of unnecessary examinations—suggest potential for substantial cost savings and reduced patient burden from contrast exposure and radiation. A formal cost-effectiveness analysis comparing the two-step strategy against usual care and comprehensive venographic screening would provide health systems with the economic evidence needed for implementation decisions.

## 5 Conclusions

### 5.1 Summary of contributions

This retrospective, single-center study of 528 patients with mild-to-moderate traumatic brain injury (TBI) who underwent dedicated venous imaging makes three principal contributions to the literature on traumatic cerebral venous sinus thrombosis (tCVST).

**First**, the study establishes that tCVST was detected in 7.8% (41 of 528) of this pre-selected imaging cohort and demonstrates that trans-sinus fracture is the dominant risk factor, with an adjusted odds ratio of 8.44 (95% CI 4.38–16.39). This finding confirms and extends prior observations from severe TBI populations to the mild-to-moderate injury spectrum.

**Second**, the study introduces a practical two-step clinical decision strategy designed for bedside application. Step 1 employs trans-sinus fracture as a binary gatekeeper (NPV 96.3%); Step 2 stratifies TF-positive patients using D-dimer concentration and headache visual analog scale score. This approach reduced the number needed to image from 13 to 3, avoiding approximately 85% of unnecessary CTV/MRV examinations.

**Third**, the study provides quantitative efficiency estimates that may inform resource allocation and represents an early, exploratory attempt to translate heterogeneous risk factors into an actionable decision tool for a clinically underspecified problem, pending external validation.

The most important takeaway from this work is that a simple, hierarchically structured two-step strategy using trans-sinus fracture as an initial gatekeeper may serve as a starting point for rationalizing CTV/MRV decisions in mild-to-moderate TBI, though its 61% sensitivity means it must augment rather than replace clinical judgment.

### 5.2 Priorities for future research

These findings are exploratory and hypothesis-generating; several priorities warrant emphasis. Prospective multicenter validation across diverse populations and imaging protocols is essential to determine whether the observed efficiency gains generalize to unselected cohorts and to establish the safety of deferring immediate venography in TF-negative patients. A dedicated investigation of the 39% of tCVST cases that occurred without trans-sinus fracture is needed to identify supplementary risk signals that could improve sensitivity without compromising the approach's simplicity. Finally, formal cost-effectiveness analysis comparing the two-step strategy against both universal screening and discretionary practice would provide health systems with the economic evidence necessary for implementation decisions.

## Data Availability

The original contributions presented in the study are included in the article/supplementary material, further inquiries can be directed to the corresponding author.
